# The Impact of Shift Work on Occupational Health Indicators among Professionally Active Adults: A Comparative Study

**DOI:** 10.3390/ijerph182111290

**Published:** 2021-10-27

**Authors:** Henrique Pereira, Gergely Fehér, Antal Tibold, Samuel Monteiro, Vítor Costa, Graça Esgalhado

**Affiliations:** 1 Department of Psychology and Education, Faculty of Social and Human Sciences, University of Beira Interior, Pólo IV, 6200-209 Covilhã, Portugal; smonteiro@ubi.pt (S.M.); vitormvc@ubi.pt (V.C.); mgpe@ubi.pt (G.E.); 2 Research Centre in Sports Sciences, Health Sciences and Human Development (CIDESD), 5001-801 Vila Real, Portugal; 3 Centre for Occupational Medicine, Medical School, University of Pécs, 7624 Pécs, Hungary; feher.gergely@pte.hu (G.F.); tibold.antal@pte.hu (A.T.); 4 NECE-Research Center in Business Science, University of Beira Interior, Pólo IV, 6200-209 Covilhã, Portugal; 5 Institute of Cognitive Psychology, Human and Social Development (IPCDHS), 3000-115 Coimbra, Portugal

**Keywords:** shift work, occupational health, burnout, work-engagement, occupational self-efficacy, depression, anxiety

## Abstract

The analysis of the impact of shift work on occupational health still needs further contributions. Therefore, we developed this research with the purpose of assessing the impact of shift work on occupational health indicators, namely burnout, work-engagement, occupational self-efficacy, and mental health functioning (symptoms of depression and anxiety), by comparing workers who did shift work (44.2% of participants) with workers who did not (55.8% of participants). A total of 695 Portuguese professionally active adults between 18 and 73 years of age (*M_age_* = 37.71; *SD* = 12.64) participated in this study and completed a survey containing a sociodemographic questionnaire and four occupational health measures: The Burnout Assessment Tool, The Work-Engagement questionnaire (UWES), The Occupational Self-Efficacy Questionnaire, and the BSI-18 for mental health symptoms. Results showed statistically significant differences (*p* < 0.05) for all indicators, demonstrating that participants who worked shifts presented lower scores of work-engagement and occupational self-efficacy, and higher scores of burnout, depression, and anxiety when compared to participants who did not work shifts. Linear regressions showed that shift work explained significant but low percentages of anxiety symptoms, low work-engagement, depression symptoms, low occupational self-efficacy, and burnout. We concluded that non-standard working hours (by shifts) are detrimental to employee occupational health, by increasing the risk of anxiety and depression levels, and burnout, and by reducing work-engagement (as a well-being indicator) and occupational self-efficacy perceptions.

## 1. Introduction

Shift work has always existed, being initially linked to protection and survival needs, such as guarding, military, or surveillance activities, having changed radically after the industrial revolution, which allowed workers to perform their duties at different times, even at night [1]. However, the nature of shift work has been changing drastically, due to economic, technological, and social reasons, to provide answers to the increase in professional activities in particular sectors such as health, transportation, communications, or security [2].

Shift work is defined by the International Labor Organization as the method of organization of working time in which workers succeed one another at the workplace so that organizations can operate longer than the hours of work of individual workers at different daily and night hours [3]. In fact, today’s societies have developed a style of productivity based on 24 h/day, 7 days a week, generating operational needs to have workers always available, through the development of a succession of different teams of workers. Thus, shift work includes night work, part-time work, and weekend work and affects a significant percentage of workers in the world, as, on average, 17% of the world population works in shifts [4]. However, the performance of shift work has important consequences for the workers’ health.

In addition to the immediate disruption of the circadian rhythm and implications for sleep quality [5], an extensive list of recent research results shows that these consequences interfere in numerous areas of human functionality, namely the following in the area of physical health: the risk of breast cancer [6], chronic fatigue, sleep problems and social jetlag [7,8,9], obesity and overweight [10], metabolic syndrome [11], carcinogenicity [12], increased cortisol secretion [13], use of psychotropic medication [14], vascular endothelial dysfunction and platelet activation [15], hypertension [16], cardiovascular mortality [17], obstructive sleep apnea [18], diabetes mellitus [19,20], women’s menopausal age and menstrual years [21], abnormal liver function [22], and overall chronic diseases [23].

Regarding consequences in mental health, many studies point to more severe effects attributable to broad binary and irregular or unpredictable shift work [24], and to gender, making women who work shifts at increased risk for poor mental health, especially depression [25], although this also affects men [26]. In fact, depression seems to be the mental disorder most influenced by shift work [27], although suicidal ideation, especially accompanied by insomnia, is also more present in shift workers [28], as well as anxiety disorders and burnout syndrome [29]. Evidence also suggests that shift work does compromise the cognitive flexibility [30] and resilience [31] of workers. The impact of shift work on mental health appears to be direct and indirect (through sleep quality) [32], with current research suggesting that sleep quality and mental health symptoms may have a bidirectional relationship [33].

When examining the consequences of shift work for occupational health, it is well-documented that it negatively impacts on job satisfaction [34,35], on the increase in occupational stress [36], on the occurrence of occupational accidents [37,38], on work-engagement [39], as a well-being indicator, lower work performance, and higher absenteeism [40,41].

The analysis of the impact of shift work still needs further studies. In the Portuguese context, the few existing studies tend to focus on specific sectors of professional activities (e.g., textile industry or transport) [42,43], leaving room for more general assessments that consider the nature of the work itself. Thus, and to fill this gap, we developed this research, where the general objective was to assess the impact of shift work on the occupational health of Portuguese workers and, more specifically, to examine differences between workers who carry out their professional activity in shifts or not in the domains of occupational burnout, work-engagement, occupational self-efficacy, and mental functioning (symptoms of depression and anxiety).

## 2. Materials and Methods

### 2.1. Measurement Instruments

As measurement instruments for this study, we used a sociodemographic questionnaire and four occupational health measures: burnout, work-engagement, occupational self-efficacy, and mental health symptoms (Table 1).

Sociodemographic information: variables included age, gender, marital status, education, place of residence, socioeconomic status, occupational status, nature of the organization they worked for, sector of activity, weekly hours of workload, length of time working, and shift work.

Burnout: The Portuguese version of the Burnout Assessment Tool (BAT) was used in this study [44]. The BAT comprises 22 items, measuring 4 fundamental symptoms of burnout: exhaustion, mental distance, emotional impairment, and cognitive impairment. Assuming that burnout is a syndrome, and therefore all four dimensions are interrelated and refer to the same underlying condition [45], we used an overall score of burnout, calculating the mean score of all items, which were rated on a five-point Likert scale ranging from 1 (never) to 5 (always). To determine overall internal reliability, a Cronbach’s α = 0.92 was obtained, demonstrating excellent internal reliability.

Work-engagement: To measure work-engagement, we used the Portuguese version of the Utrecht Work-Engagement Scale (UWES) [46]. Work-engagement is defined as a positive, fulfilling, work-related state of mind characterized by vigor, dedication, and absorption. The Portuguese version used here consists of 9 items, rated on a 7-level response scale ranging from 1 (never) to 7 (always). Examples of items include: “I am enthusiastic about my work”, or “At my work, I feel strong and vigorous”. The total score of work-engagement was calculated using a simple mean score, with higher scores indicating more work-engagement. To determine overall internal reliability, a Cronbach’s α = 0.94 was obtained, demonstrating excellent internal reliability.

Occupational Self-Efficacy: We used the short version of the Occupational Self-Efficacy scale [47], consisting of 6 items, as it assesses the confidence or the belief an individual has in her or his ability to cope with difficult tasks or problems within an occupational environment. Items were translated to Portuguese following existing guidelines for the translation of research instruments [48]. Items are rated on a six-level response scale ranging from 1 (not at all true) to 6 (completely true). High values reflect high occupational self-efficacy. Examples of items include: “I can remain calm when facing difficulties in my job because I can rely on my abilities”, or “When I am confronted with a problem in my job, I can usually find several solutions”. A total score of occupational self-efficacy was calculated using a simple mean score, with higher scores indicating higher levels of self-efficacy. To determine overall internal reliability, a Cronbach’s α = 0.89 was obtained, demonstrating excellent internal reliability.

Mental Health Symptoms (Depression and Anxiety): Mental health symptoms were measured using the anxiety and depression sub-scales of the Portuguese version of the Brief Symptom Inventory (BSI-18) [49]. This is an 18-item scale that measures depression, anxiety, and somatic symptoms in nonclinical populations and is beneficial to screen mental health symptoms. For this study, only the depression and anxiety subscales (6 items each) were used since they represent the most prevalent mental health problems among the general population. Participants answered questions based on their self-assessment of mental symptoms using a 5-point scale ranging from 0 (never) to 4 (always). A total score was calculated using a simple mean score, with higher scores indicating more symptoms. Cronbach’s alphas revealed high reliability levels for each factor (0.89 for depression, 0.87 for anxiety). To determine overall internal reliability, a Cronbach’s α = 0.93 was obtained, demonstrating excellent internal reliability.

### 2.2. Procedures

Data collection was carried out via the internet and participants were recruited through a website created for this purpose between October and December 2020. Participation was voluntary and did not involve any monetary gratification. Upon receiving the research link, the site informed participants about the research objectives, filling instructions, informed consent, and researchers’ contacts. A total of 3860 notifications were sent, and 695 participants responded favorably and answered the survey correctly (response rate of 18%). This research met all ethical criteria, including informed consent, anonymity, and confidentiality. Inclusion criteria were being professionally active, being Portuguese, being able to read Portuguese, and being over 18 years of age. This study was approved by the Ethics Committee of the University of Beira Interior (Portugal) (CE-UBI-PJ-2020-088).

### 2.3. Data Analysis

Descriptive statistics were performed to describe the sample (mean, standard deviation, frequencies, and percentages). The normality of the data was evaluated and confirmed by the Kolmogorov–Smirnov test. To assess differences between the comparison groups (shift work, yes/no), Student’s t-distribution tests were conducted. To assess the association between variables, Pearson’s correlation coefficients were calculated, and to assess the predictive power of shift work on occupational health indicators, we performed linear logistic regression analyses. To measure internal consistency, we used Cronbach’s alpha, and all scores for each measurement instrument are presented in Table 2. To avoid Type I errors, we conducted Bonferroni correction tests. To measure multicollinearity, we used the variance inflation factor (VIF = 1), which indicated that the variables were not correlated. All statistical procedures were conducted using the Statistical Package for the Social Sciences (SPSS version 27, IBM Corporation, Armonk, New York, USA).

## 3. Results

A total of 695 Portuguese professionally active adults, between 18 and 73 years of age (*M_age_* = 37.71; *SD* = 12.64), participated in the study. The majority (58.7%) identified as female, were single (44%), had a university education (76.4%), resided in urban settings (83.3%), and stated belonging to the middle socioeconomic status (55.4%). Regarding their occupational status, the majority indicated that they were employed (59.9%), worked for public organizations (52.1%), and worked in the tertiary sector (89.6%) for an average of 10.26 years, working for 39.07 h (on average) per week. Regarding shift work, 55.8% of participants said that they did not do shift work. Table 3 shows the sociodemographic characteristics in detail.

We analyzed differences in occupational health indicators (burnout, work-engagement, occupational self-efficacy, and mental health symptoms) by shift work. Results showed statistically significant differences (*p* < 0.05) for all indicators, namely: burnout (t_(686)_ = −2.260; *p* = 0.024), work-engagement (t_(685)_ = 4.583; *p* < 0.001), occupational self-efficacy (t_(680)_ = 3.680; *p* < 0.001), depression symptoms (t_(662)_ = −3.827; *p* < 0.001), and anxiety symptoms (t_(663)_ = −4.946; *p* < 0.001), indicating that participants who worked shifts presented lowers scores of occupational health (see Table 4 for further details).

We also created a correlation matrix to assess the levels of association between occupational health indicators. As shown in Table 5, significant and strong correlations were found for all variables.

Finally, we conducted five logistic regressions for shift work predicting each occupational indicator. As displayed in Table 6, all models are significant and shift work explained 4% of anxiety symptoms, 3% of low work-engagement, 2% of depression symptoms, 2% of low occupational self-efficacy, and 1% of burnout.

## 4. Discussion

The current paper explored the relation between shift work and burnout, work-engagement, occupational self-efficacy, and depression and anxiety symptoms. Shift work, as a specific human-work modality, with specific potential occupational hazards, was evidenced in its differences to the normal and regular working situation.

Previous published research had reviewed various impacts of shift work on health indicators [50] and chronic health conditions [23]. Smolensky et al. [51] mention that non-standard permanent and rotating night work schedules require reorganizing the circadian time structure to the unnatural routine of nocturnal activity and diurnal sleep. Hence, the negative effects of shift work on physical health and performance are primarily due to disturbances in the normal circadian rhythm [52]. The deterioration in occupational and mental health indicators will probably be associated with the same genesis, worsened by interpersonal, social, and communitarian difficulties to a life organization de-tuned from the collective mainstream.

In our study, these behavioral changes, which are, in fact, life inversion strategies required by work schedules and organizational routines, are shown to introduce statistically significant relevant differences compared to the usual work time schedules, particularly in the specific prism of occupational and mental health variables and considered indicators. Workers who worked in shifts had a higher prevalence of negative/hazard factors and less evidence of positive/protective factors. Results showed that those who do shift work presented higher values of burnout, depression, and anxiety symptoms than those who did not do shift work.

The sub-sample of shift workers also displayed lower values of work-engagement and occupational self-efficacy. That is, participants in the current study that did shift work, when compared to workers that did not do shift work, presented worse scores in a series of occupational health and mental health indicators. Moreover, working in shifts significantly predicted increased levels of burnout, a decrease in work-engagement and occupational self-efficacy, and an increase in depression and anxiety symptoms. Therefore, findings in the current study are aligned with those of recent systematic reviews and meta-analytic studies [24,25,53,54] that demonstrate a relation between shift work and poorer occupational health.

Our findings may present multilevel implications, for those responsible for managing the work time and schedules in organizations, and for workers as well, as they should be more aware of the work heterogeneity, work demands/risks, and potential impacts. Additionally, for those who define labor policies and legislative regulations, as reinforced by our results, tailored demands at the occupational health level should be taken into consideration, such as specific benefits and associated rewards, and specific regulation in terms of occupational health monitoring and intervention rules. Shift work will require an occupational health preventive approach distinct or nonstandard from regular work, and, from our results, a more frequent screening of the workers’ well-being and mental health indicators should be considered.

The highlighted comparative differences reinforce the importance of a shift work double evaluation—risk assessment and effects/impacts evaluation. Our results, in terms of the potential deterioration of occupational health indicators and the subsequent potential health-related productivity loss, are in line with other results [52].

### Limitations and Future Directions

Results from the current study are not without limitations. Shift work was measured by a dichotomous item, which may not profusely capture all the diversity associated with shift work (e.g., night/evening work, weekend work, irregular/unpredictable work schedules). The study is cross-sectional and therefore considers respondents’ perceptions on a single moment in time, which does not allow a further explanation of the eventual causal relationships. In this case, data collected concurred with the COVID-19 pandemic, which might have contributed to how participants evaluated their mental and occupational health. Finally, the use of a convenience sample hinders the generalization of the results.

Additional and future research is needed to understand how shift work impacts on work-engagement, and other positive psychology constructs. Further research should also analyze different subtypes of shift work, should explore different populations, and different sociodemographic segments, and the role of intervening variables, such as sleep quality, in the relationship between shift work and affective disorders and occupational health.

## 5. Conclusions

We conclude that non-standard work hours (by shifts) are detrimental to employee occupational health, by increasing the risk of anxiety and depression levels, by increasing burnout, and by reducing work-engagement (as a well-being indicator) and occupational self-efficacy perceptions. Therefore, the indicators evaluated and used in this study are discriminative, and a valid example of occupational indicators that may be considered in the shift work risk/effect assessment in work environments. By exploring positive outcomes, our results suggest that, in our sample, increasing employees’ work-engagement and occupational self-efficacy might reduce burnout and depressive and anxiety symptoms, independently of working or not working in shifts.

Additionally, the current study presents a valuable contribution to exploring shift work’s impact on occupational health indicators, adding to a growing body of literature that supports the link between shift work and poorer health in general, and occupational and mental health domains in particular. In addition to these established and well-studied relationships, it is one of the few studies that explores the impact of shift work on work-engagement and occupational self-efficacy, providing some innovative contributions to the positive occupational health field.

## Figures and Tables

**Table 1:** Variables and considered dimensions.

Variables	Dimensions
Burnout	Exhaustion, Mental distance, Emotional impairment, Cognitive impairment.
Work-Engagement	Vigor, Dedication, Absorption.
Occupational Self-Efficacy	Occupational Self-Efficacy.
Mental Health Symptoms	Depression, Anxiety.

**Table 2:** Cronbach’s alphas for all measurement instruments.

Instrument	Cronbach’s Alpha
Burnout Assessment Tool	0.92
Work-Engagement	0.94
Occupational Self-Efficacy	0.89
Mental Health Symptoms	0.93

**Table 3:** Sociodemographic characteristics of the participants (*n* = 695; *M_age_* = 37.71; *SD* = 12.64; *M_workload_* = 39.07 weekly hours; *SD* = 32.77; *M_length_* = 10.26 years; *SD* = 10.40).

		*n*	%
Gender	Male	284	40.9
	Female	408	58.7
	Other	3	0.4
Marital Status	Single	305	44.0
	Married	238	34.2
	Civil Union	92	13.2
	Divorced	53	7.6
	Widower	7	1.0
Education	Up to 9 years of school	19	2.8
	Up to 12 years of school	144	20.8
	BA	159	22.9
	MA	234	33.6
	PhD	139	19.9
Place of residence	Small rural	83	12.0
	Large rural	33	4.7
	Small urban	278	40.0
	Large urban	301	43.3
Socioeconomic status	Low	28	4.0
	Low-Middle	165	23.7
	Middle	385	55.4
	High-Middle	104	15.0
	High	13	1.9
Occupational Status	Working student	188	27
	Self-employed	76	10.9
	Employed	416	59.9
	Retired but active	15	2.2
Nature of the organization they work for	Public	362	52.1
	Private	304	43.8
	Mixed	222	3.2
	Other	6	0.9
Sector of activity	Primary	17	2.4
	Secondary	55	8.0
	Tertiary	623	89.6
Shift Work	No	388	55.8
	Yes	307	44.2

**Table 4:** Results for Burnout, Work-Engagement, Occupational Self-Efficacy, and Mental Health Symptoms by shift work.

Variable	Shift Work	*M* (*SD*)	*t*(*df*)	*p*
Burnout	No	2.37 (0.60)	−2.260(686)	0.024 *
	Yes	2.48 (0.69)		
Work-Engagement	No	4.65 (1.30)	4.583(685)	0.000 **
	Yes	4.18 (1.41)		
Occupational Self-Efficacy	No	3.66 (0.81)	3.680(680)	0.000 **
	Yes	3.42 (0.89)		
Depression	No	0.94 (0.84)	−3.827(662)	0.000 **
	Yes	1.21 (0.95)		
Anxiety	No	0.93 (0.73)	−4.946(663)	0.000 **
	Yes	1.24 (0.92)		

* *p* < 0.05; ** *p* < 0.001.

**Table 5:** Correlation matrix.

	1	2	3	4	5
1—Burnout	1				
2—Work-Engagement	−0.585 **	1			
3—Occupational Self-Efficacy	−0.465 **	0.569 **	1		
4—Depression	0.744 **	−0.519 **	−0.418 **	1	
5—Anxiety	0.721 **	−0.411 **	−0.397 **	0.798 **	1

** *p* < 0.001.

**Table 6:** Logistic regression for shift work predicting occupational health indicators.

	*B*	*SE*	*β*	*R* ^2^	*F*
Burnout	0.112	0.049	0.086 *	0.01	5.105 *
Work-Engagement	−0.475	0.104	−0.172 **	0.03	21.003 **
Occupational Self-Efficacy	−0.240	0.065	−0.140 **	0.02	13.546 **
Depression	0.267	0.070	0.147 **	0.02	14.645 **
Anxiety	0.316	0.064	0.189 **	0.04	24.466 **

* *p* < 0.05; ** *p* < 0.001.

## Data Availability

The data presented in this study are available upon request.
